# Low-dose cryo electron ptychography via non-convex Bayesian optimization

**DOI:** 10.1038/s41598-017-07488-y

**Published:** 2017-08-29

**Authors:** Philipp Michael Pelz, Wen Xuan Qiu, Robert Bücker, Günther Kassier, R. J. Dwayne Miller

**Affiliations:** 1 Max Planck Institute for the Structure and Dynamics of Matter, Center for Free Electron Laser Science, Luruper Chaussee 149, 22761 Hamburg, Germany; 2 Department of Physics, University of Hamburg, Hamburg, 22761 Germany; 3 Departments of Chemistry and Physics, University of Toronto, 80 St. George Street, Toronto, M5S 1H6 Canada

## Abstract

Electron ptychography has seen a recent surge of interest for phase sensitive imaging at atomic or near-atomic resolution. However, applications are so far mainly limited to radiation-hard samples, because the required doses are too high for imaging biological samples at high resolution. We propose the use of non-convex Bayesian optimization to overcome this problem, and show via numerical simulations that the dose required for successful reconstruction can be reduced by two orders of magnitude compared to previous experiments. As an important application we suggest to use this method for imaging single biological macromolecules at cryogenic temperatures and demonstrate 2D single-particle reconstructions from simulated data with a resolution up to 5.4 Å at a dose of 20*e* ^*−*^/Å^2^. When averaging over only 30 low-dose datasets, a 2D resolution around 3.5 Å is possible for macromolecular complexes even below 100 kDa. With its independence from the microscope transfer function, direct recovery of phase contrast, and better scaling of signal-to-noise ratio, low-dose cryo electron ptychography may become a promising alternative to Zernike phase-contrast microscopy.

## Introduction

The advent of direct electron detectors has led to a resolution revolution in the field of cryo electron microscopy in the last few years. The technique is now producing three-dimensional atomic potential maps of biological macromolecules of a few 100 kDa or lower with a resolution better than 3.5 Å^1–3^, such that individual amino acid side-chains can be resolved. An important role in this revolution play new image processing algorithms based on a Bayesian approach, which infer important parameters without user intervention^4^. Also the correction of beam-induced motion has become possible mostly due to the new generation of detectors^5, 6^. However, several challenges remain to be overcome in order to routinely reach 3 Å resolution also for small complexes^7, 8^: Firstly, beam-induced specimen charging and subsequent motion currently still render the high resolution information of the first few frames of a high repetition rate movie recorded with a direct electron detector unusable^9^, because the motion is too fast to efficiently correct for it. Secondly, the Detective Quantum Efficiency (DQE) of detectors is still imperfect at high spatial frequencies^8, 10^. Thirdly, the contrast of single images can still be improved to enable reconstructions with fewer particles and increase the throughput^10^.

The last of these challenges has recently been addressed with a new phase plate model^11, 12^, which is comparatively simple to use and provides excellent contrast at low spatial frequencies. In addition to this hardware-based approach to achieve linear phase contrast in the measured amplitudes, discovered by Zernike in the 1930s^13^, it is also possible to algorithmically retrieve the phase information from a set of coherent diffraction measurements. One such technique, commonly known as *ptychography* or *scanning coherent diffractive microscopy* ^14^, is becoming increasingly popular in the field of materials science due to experimental robustness and the possibility to obtain quantitative phase contrast over an essentially unlimited field of view^15, 16^. The use of ptychography for imaging radiation sensitive samples with electrons at high resolution is however precluded so far by its high dose requirements.

Here, we show how the use of non-convex Bayesian optimization to solve the ptychographic phase retrieval problem fulfills the dose requirements for imaging biological macromolecules and makes it possible to obtain 2D images from single particles with sub-nanometer resolution. After a short introduction into the technique, we will also mention how ptychography offers improvements for the other two challenges discussed above.

Despite being initially proposed as a solution to the phase problem for electrons^17, 18^, ptychography has seen its biggest success in X-ray imaging, due to the less stringent sample requirements and the experimental need for lensless imaging techniques. Recent developments include the introduction of iterative algorithms to enable the reconstruction of datasets collected with an out-of focus probe^19, 20^, which decreases the memory requirements of the method dramatically. The algorithms also have the capability for the correction of experimental difficulties such as unknown scan positions^21–23^, partial coherence^24^, probe movement during exposure^25, 26^, intensity fluctuations during the scan^24, 27^ and reconstruction of background noise^15, 27^.

In recent years, some of these advances have been applied in the context of electron microscopy and yielded atomic resolution reconstructions of low-atomic number materials^28–30^ and quantitative phase information^15^.

**Figure 1:**
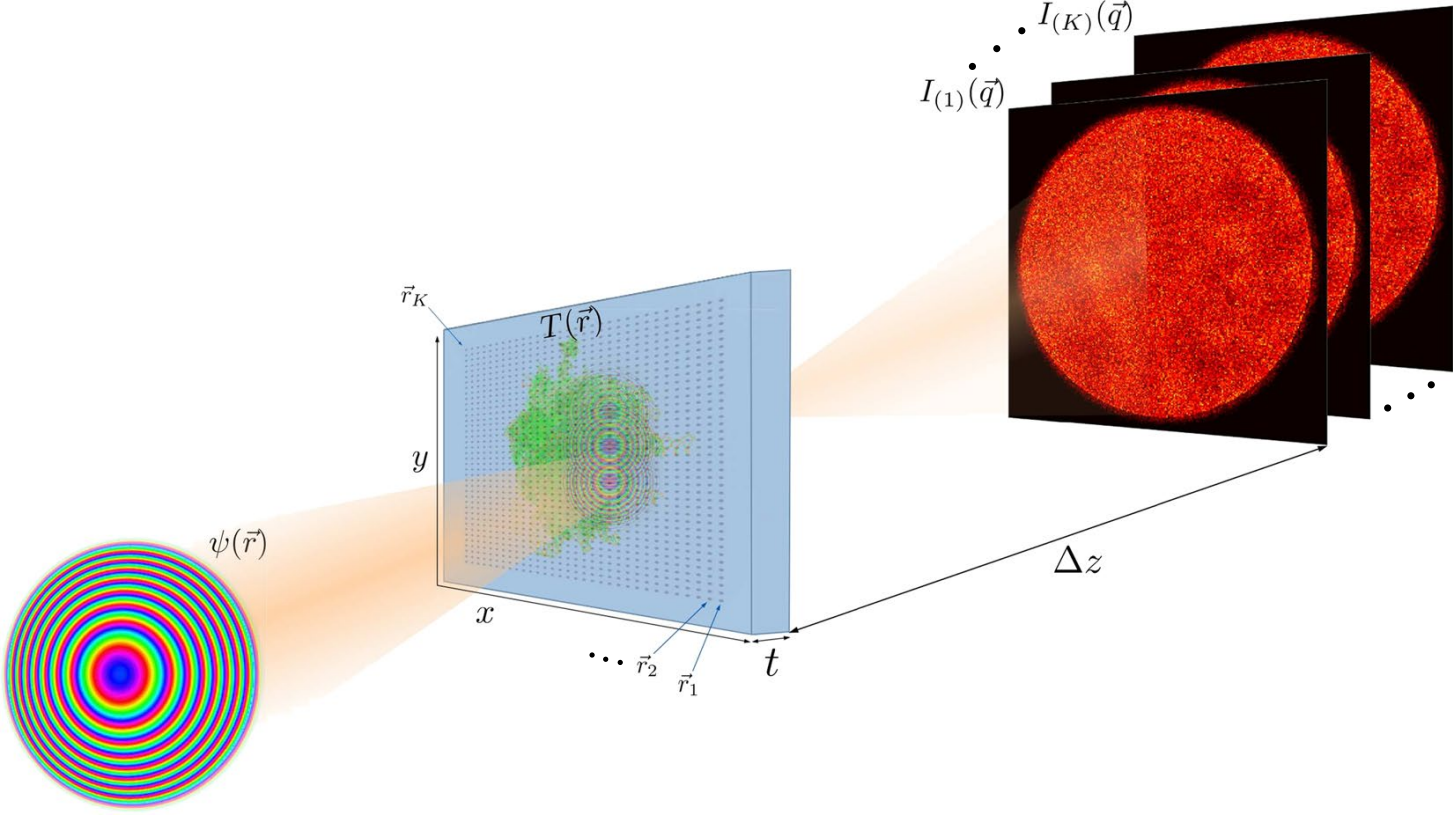
Experimental geometry in ptychography. The coherent electron wave function $\psi (\vec{r})$ illuminates several regions (centered at ${\vec{r}}_{{\rm{1}}\ldots {\rm{K}}}$) across the sample, which is characterized by the transmission function $T(\vec{r})$. For each position, a 2D diffraction pattern ${I}_{(1\ldots K)}$ is recorded in the far field at distance Δ*z*. The sample thickness *t* can be neglected for biological macromolecules in the reconstruction at the resolutions presented in this paper.

Figure 1 shows the experimental set-up for an out-of focus ptychography experiment. A ptychographic dataset is collected by scanning a spatially confined, coherent beam, subsequently called ‘probe’, over the specimen and recording far-field diffraction patterns at a series of positions such that the illuminated regions of neighboring positions overlap. The diffraction-limited resolution *r*_d_ (half-period) of the final image is given by ${r}_{{\rm{d}}}=\frac{\lambda \cdot {\rm{\Delta }}z}{{N}_{{\rm{pix}}}\cdot {d}_{{\rm{pix}}}}$, where *N*_pix_ is the number of detector pixels, *d*_pix_ is the detector pixel size and *λ* is the de-Broglie-wavelength of the electrons. Given the set of positions ${\vec{r}}_{{\rm{1}}\ldots {\rm{K}}}$ and a realistic forward model for the formation of the corresponding diffraction patterns ${I}_{(1\ldots K)}$, the complex-valued transmission function $T(\vec{r})$, which describes the atomic properties of the specimen^31^, can then be retrieved by solving a non-convex inverse problem. The electron dose used for successful reconstruction has exceeded 1 × 10^3^ *e* ^−^/Å^2^ so far, limiting the usability of ptychography to radiation-hard specimens. Table 1 lists recently published electron ptychography experiments and the used average electron doses. The lowest dose was reported in ref. 32, which used an estimated 3.33 × 10^3^ *e* ^−^/Å^2^ at a resolution of d_r_ = 58.4 pm, resulting in a dose of 1.1 × 10^3^ *e* ^−^/pixel and achieving a line resolution of 2.3 Å, demonstrated by resolving the lattice spacing of gold nanoparticles. We will demonstrate via simulations that it is possible to reduce this dose by a factor of 100, thus reaching the dose range allowed for imaging biological macromolecules.

**Table 1:** List of previously published electron ptychography experiments and used electron dose.

Reference	resolution	*e* ^−^/Å^2^
D’Alfonso *et al*.^30^	~1.5 Å	1.77 × 10^4^
Yang *et al*.^29^	atomic	1.3 × 10^4^
Putkunz *et al*.^28^	~1 Å	9.2 × 10^6^
Humphry *et al*.^32^	~2.3 Å	3.33 × 10^3^

The problem of beam-induced sample movement has already been addressed before the development of fast direct detectors. Scanning with small spots of several 10 nm in size over a vitrified sample has shown to reduce beam induced specimen movement^33–35^ in real-space imaging and it has been noted that the remaining movement may be due to radiation damage, not sample charging^33^. Ptychography naturally operates with a confined beam, thus minimizing the area where charge can build up, such that the movement should be reduced compared to the illumination of large areas in cryo-EM. The sampling requirements given by the experimental setup allow to operate the detector with large effective pixel sizes, such that the DQE and MTF are at near-perfect values and can be neglected in the reconstruction. The overhead resulting from the need to take multiple exposures can therefore be greatly reduced by using fast detectors with few pixels.

## Results

### Image formation of cryo-TEM and ptychography

We perform multislice simulations of three different biological macromolecules with molecular weights ranging from 64 kDa to 4 MDa. We choose the 64 kDa hemoglobin^36^, the 706 kDa 20S proteasome from yeast^2^, and the 4 MDa human ribosome^37^. We create atomic potential maps using the Matlab code InSilicoTem^38^, with a thickness of 50 nm and at an electron energy of 300 keV. We use the isolated atom superposition approximation, without solving the Poisson-Boltzmann equations for the interaction between the molecule and the ions. We also do not model the amorphousness of the solvent, which was performed in ref. 38 using molecular dynamics simulations, but was seen to have a negligible effect at very low doses. As described in ref. 38, we model the imaginary part of the potential via the inelastic mean free path, creating a minimal transmission contrast between the vitreous ice and the protein. Using these potential maps, we simulate a ptychography experiment by cropping three-dimensional slices from the potential at several positions and propagate a coherent incoming wave through the slices using the methods described in ref. 39 in custom code. The final model for the formation of the intensity on the detector is then

1 $${I}_{0}(\vec{q})={| {\mathcal F} [{\psi }_{exit}(\vec{r})]|}^{2}$$

for the diffraction pattern and

2 $${I}_{0}(\vec{q})={|{ {\mathcal F} }^{-1}[ {\mathcal F} [{\psi }_{exit}(\vec{r})]\cdot {\rm{CTF}}(\vec{q})]|}^{2}$$

for the cryo-EM image, where ${\mathcal F}$ and ${ {\mathcal F} }^{-1}$ denote the forward and inverse Fourier transform, and CTF the contrast transfer function, respectively.

The detector properties are modeled as in ref. 38, by multiplying the Fourier transform of the exit-wave intensity with the square root of the detective quantum efficiency $\sqrt{{\rm{DQE}}(\vec{q})}$ ^40^, before applying shot noise by sampling from a Poissonian distribution. We finally convolve the noisy signal with the noise transfer function ${\rm{NTF}}(\vec{q})$ to yield the measured intensity.

A notable difference both in simulation and practice is the fact that for cryo-EM, usually no pixel binning is applied to maximize the imaged area and increase throughput. Therefore, also high spatial-frequency regions with low values of DQE and NTF are used for image formation^41^. For ptychography, on the other hand, the detector can be heavily binned, as long as the real-space patch given by $\lambda {\rm{\Delta }}z/{d}_{{\rm{pix}}}\equiv {r}_{{\rm{d}}}\cdot {N}_{{\rm{pix}}}$ still encompasses the probe beam on the sample. For typical detectors this condition is fulfilled at bin sizes equivalent to a few percent of the Nyquist frequency. This leads to a near-constant DQE and a near-unity NTF, such that they can be omitted in the ptychography reconstructions, whereas we still include them in the simulation of the diffraction data. We note, however, that a convolution with a detector transfer function can be modeled with a partially coherent beam if necessary, as demonstrated in refs 42 and 43. We choose the Gatan K2 Summit as the detector for our simulations because it has the highest published DQE and MTF values at low spatial frequencies at 300 keV^41^. We note that direct detection cameras with frame rates of 1 kHz and above may be more suitable for high-throughput scanning experiments^44–46^, but characteristics for these cameras at 300 keV are either not published or inferior to the K2 Summit. Assuming the K2 Summit for both ptychography and phase-contrast TEM simulations also simplifies a direct comparison between the two methods. The intensity after detection is modeled as^38^:

3 $$I(\vec{q})={ {\mathcal F} }^{-1}[ {\mathcal F} \,[{\rm{Poisson}}({ {\mathcal F} }^{-1}[ {\mathcal F} \,[{I}_{0}(\vec{q})]\cdot \sqrt{{\rm{DQE}}(\vec{q})}])]\cdot {\rm{NTF}}(\vec{q})],$$

where NTF and DQE are properties of the detector^40, 45^ and Poisson(*x*) samples from a Poisson distribution with mean *x*.

### Single-particle reconstruction

**Figure 2:**
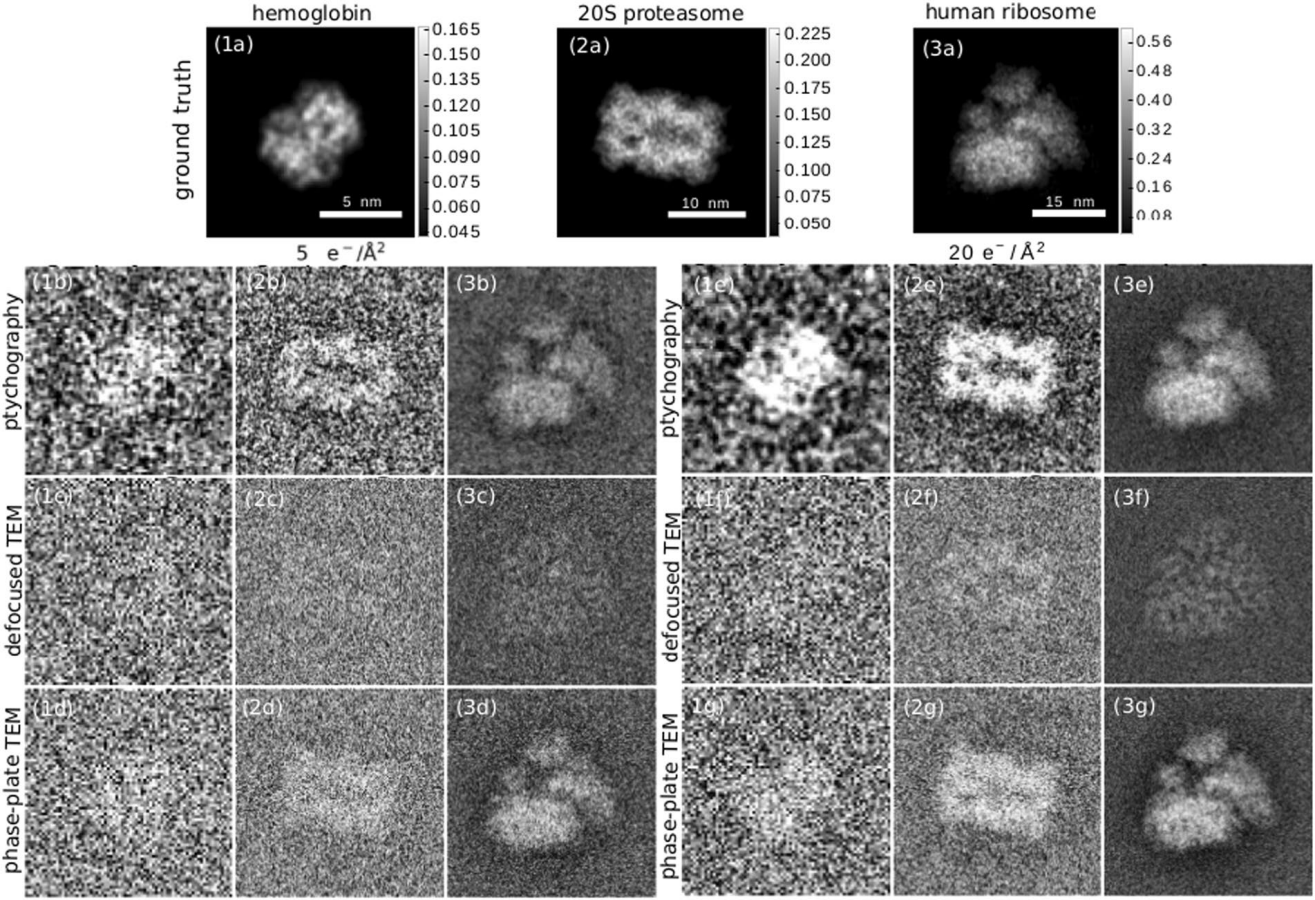
Cryo-electron ptychography reconstructions from simulated data and simulated cryo-EM images for different doses and 3 macromolecules with growing molecular weights in columns 1–3. Row (**a**) Phase of the transmission function, the ground truth for the ptychography reconstructions. The scale bar next to the figures is in rad. Rows (**b**) and (**e**) ptychography reconstruction at doses of 5 *e* ^−^/Å^2^ and 20 *e* ^−^/Å^2^. Rows (**c**) and (**f**) Simulated cryo-EM image with a defocus of 1.6 μm at a dose of 5 *e* ^−^/Å^2^ and 20 *e* ^−^/Å^2^. Rows (**d**) and (**g**) Simulated cryo-EM image with a Zernike phase plate and a defocus of 50 nm at doses of 5 *e* ^−^/Å^2^ and 20 *e* ^−^/Å^2^. Column (1) hemoglobin, column (2) 20S proteasome, column (3) human ribosome.

**Figure 3:**
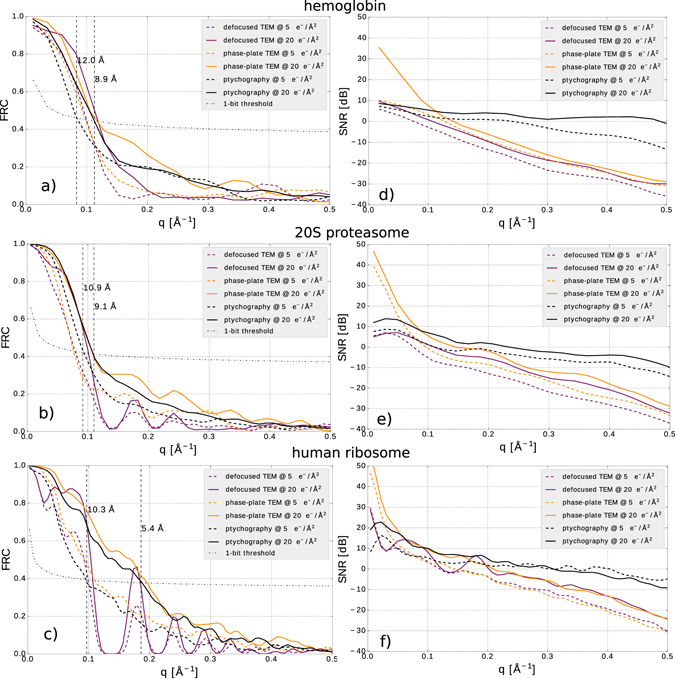
FRC (**a**)–(**c**) and SNR (**d**)–(**f**) as a function of spatial frequency for the cryo-electron ptychography reconstructions and simulated cryo-EM images in Fig. 2.

Figure 2 shows a comparison of low-dose ptychography reconstructions with currently used methods for single-particle imaging with electrons: defocus-based cryo-EM, and Zernike phase contrast cryo-EM with a Volta phase-plate. We choose exemplary doses of 5 *e* ^−^/Å^2^ as the typical threshold where the highest resolution details are destroyed^47^ and 20 *e* ^−^/Å^2^ as a typical dose at which experiments are performed. We have reversed the contrast in the cryo-EM images to simplify the visual comparison with the ptychography reconstructions. To quantitatively assess the image quality, we have computed the 2D Fourier Ring Correlation (FRC)^48^ with the ground truth for the both ptychographic reconstruction and simulated cryo-EM images of the macromolecules, as shown in Fig. 3. As ground truth for the images we use the electron counts in a noiseless, aberration-free phase-plate image. Using the 1-bit criterion as a resolution threshold^48^, the achieved resolutions at 5 *e* ^−^/Å^2^ and 20 *e* ^−^/Å^2^, respectively, are 12 Å and 8.9 Å for hemoglobin; 10.9 Å and 9.1 Å for 20S proteasome; and 10.3 Å and 5.4 Å for human ribosome. In the case 20S proteasome, these values are identical to the FRC threshold for the phase plate image; for hemoglobin and human ribosome, the phase plate image yields a slightly better resolution of 8.7 Å and 5.1 Å respectively at a dose of 20 *e* ^−^/Å^2^ and 10 Å at a dose of 5 *e* ^−^/Å^2^. As the FRC is insensitive to very small and very large values of signal-to-noise ratio (SNR), we also show the spatial-frequency resolved SNR in Fig. 3(d)–(f). We define the SNR as

4 $$SNR(q)=10\cdot {\mathrm{log}}_{{\rm{10}}}(\frac{{| {\mathcal F} [T(\vec{r})]|}^{2}}{{| {\mathcal F} [T(\vec{r})]- {\mathcal F} [{T}_{model}(\vec{r})]|}^{2}}){\rm{dB}}.$$

The SNR of the ptychographic reconstruction is significantly lower than the SNR of the phase-plate image for all three particles at spatial frequencies below 0.1 Å^−1^. It does however scale better to high spatial frequencies, and does not drop below −15 dB for all particles and all resolution up to nearly 0.5 Å^−1^, where the SNR is close to two orders of magnitude better than the phase-plate image SNR. This helps ptychography perform better when multiple reconstructions are averaged, because a positive single-digit SNR can be reached with fewer particles.

### Effect of averaging

In single-particle cryo-EM, a three-dimensional reconstruction can be obtained by collecting a large ensemble of 2D images, before orienting and averaging them in three dimensions, such that a satisfactory SNR is achieved. A similar 3D reconstruction from ptychographic data is out of the scope of this paper. A straightforward approach would be to use the reconstructed 2D phase images as an input to existing 3D reconstruction algorithms^4, 49^, as is done routinely in ptychographic X-ray tomography^50^; however, it is likely that a dedicated algorithm which reconstructs the 3D structure directly from the diffraction patterns will achieve the best results. Coarse orientation alignment for such an algorithm could be done in real space from 2D reconstructions shown here. We leave the evaluation of such approaches to future studies, and for now concentrate on the achievable 2D resolution when averaging a larger ensemble of particles.

**Figure 4:**
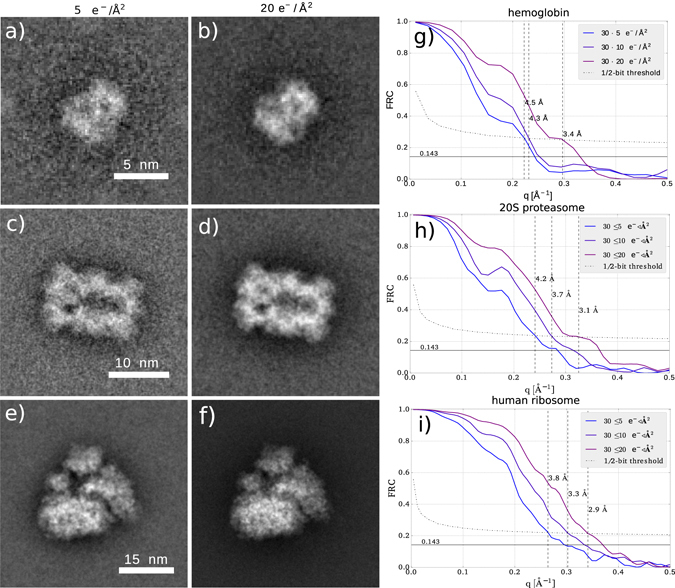
Average over 30 ptychographic reconstructions from independent data sets for (**a**) hemoglobin with 5 *e* ^−^/Å^2^, (**b**) hemoglobin with 20 *e* ^−^/Å^2^, (**c**) proteasome 20S with 5 *e* ^−^/Å^2^, (**d**) proteasome 20S with 20 *e* ^−^/Å^2^, (**e**) human ribosome with 5 *e* ^−^/Å^2^, (**f**) human ribosome with 20 *e* ^−^/Å^2^. FRC of averaged reconstructions from independent data sets for (**g**) hemoglobin, (**h**) proteasome 20S (**i**) human ribosome.

To give a rough estimate how the resolution and SNR achieved by our algorithm scales with averaging over multiple datasets, we simulate 30 independent datasets with identically oriented particles, and average the reconstruction results. We found that the resolution corresponding to the diffraction limit as defined by the probe-forming aperture is achieved with roughly 40 averaged reconstructions (see the Supplementary Information). Superresolution beyond this point is in principle possible, but due to the low electron counts and the low contrast transfer for higher angles, more images are needed for further improvements. To limit the amount of necessary computation we use an average of 30 images, where a resolution of roughly 3 Å is reached, close to the resolution corresponding to the probe-forming aperture of 2.7 Å. Figure 4(a)–(f) show images of the averaged reconstructions of the three samples, at doses of 5 *e* ^−^/Å^2^ and 20 *e* ^−^/Å^2^ respectively. We also compare the FRC between respectively 30 averaged reconstructions of 60 independently created ptychographic data sets, to give a resolution estimate. We use here the 1/2-bit resolution threshold discussed in ref. 48, which gives a slightly more conservative estimate than the 0.143-criterion commonly used for the 3D Fourier Shell correlation in averaged reconstructions for 3D cryo-EM. With averaging, a resolution of 3.4 Å is achieved for hemoglobin, 3.1 Å for 20S proteasome and 2.9 Å for human ribosome, all at a dose of 20 *e* ^−^/Å^2^. This shows that cryo-electron ptychography can recover atomic resolution information in 2D from only tens of averaged images, facilitated by the favorable scaling of the SNR with spatial frequency, as discussed above. It may therefore be possible to drastically reduce the number of particles that is required for a successful 3D reconstruction at atomic resolution. A rough tomographic estimate^51^ suggests that the ribosome could be reconstructed to 3 Å resolution in 3D with less than 10000 particles.

### Probe and dose dependence

**Figure 5:**
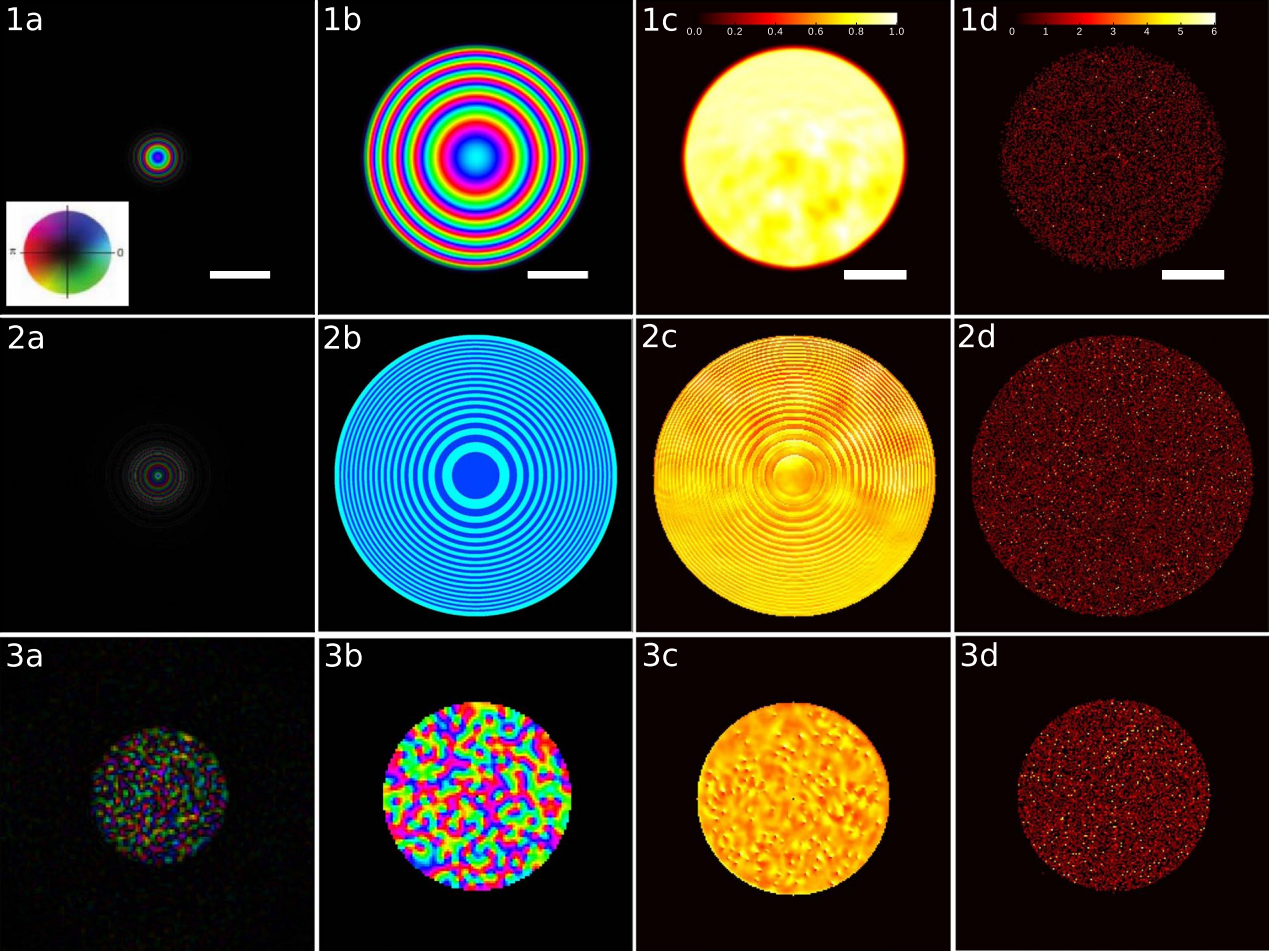
Different probes evaluated in this paper and corresponding diffraction patterns. Row 1: defocused beam with defocus aberration of 400 nm, convergence half-angle 9.2 mrad; row 2: defocused beam created by a Fresnel zone plate, 600 nm from focus; row 3: randomized beam, generated by a holographic phase plate and focused by a conventional lens. Column (**a**) beam in real space, at the sample position, scale bar is 8.5 nm; column (**b**) beam at the probe forming aperture, scale bar is 4.5 mrad; column (**c**) diffraction pattern of human ribosome at unlimited dose, normalized to the maximum intensity; column (**d**) diffraction pattern for a scan with an electron dose of 20 *e* ^−^/Å^2^. The inset in 1a shows the color wheel that is used to represent amplitude and phase in columns (**a**) and (**b**).

It is well-known that the phase profile of the ptychographic probe can heavily influence the reconstruction quality^52–56^. Here, we numerically test three different probes, depicted in Fig. 5, and their influence on the reconstruction SNR at low and high doses: (1) a standard defocused probe with defocus aberration of 400 nm, (2) a defocused Fresnel Zone Plate (FZP), and (3) a randomized probe generated by a holographic phase plate and a conventional lens. Figure 5 depicts these probe in real and Fourier space, and typical diffraction patterns at infinite dose and low dose. The FZP was recently suggested as a phase modulator for bright-field STEM^57^, because its simple phase modulation allows analytical retrieval of linear phase contrast. However, diffractive optics typically have imperfections due to the manufacturing process, which introduce errors and dose inefficiency if the phase modulation is obtained by a simple fitting procedure. Iterative ptychography algorithms allow for the simultaneous retrieval of the probe wave function^19, 20^, and therefore offer full flexibility in the design of the phase profile. Empirically, probes with a diffuse phase profile result in better reconstructions; therefore, we test as a third probe a random illumination generated by a holographic phase plate and a focusing lens.

**Figure 6:**
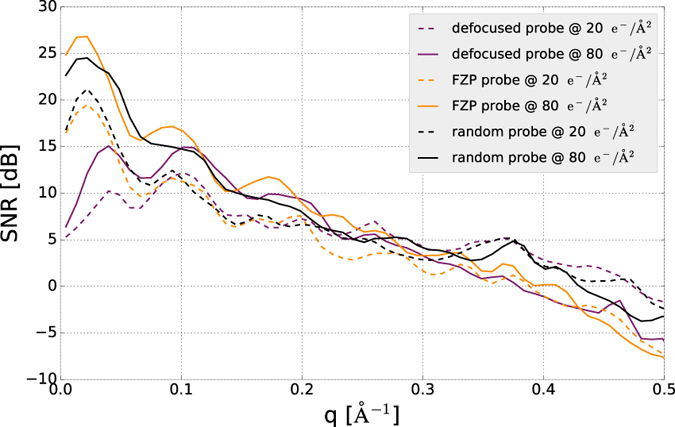
SNR of reconstructions of the human ribosome at different radiation doses using the defocused probe, the Fresnel zone plate and the randomized probe.

Figure 6 shows the SNR of the three proposed probes as a function of spatial frequency for doses of 20 *e* ^−^/Å^2^ and 80 *e* ^−^/Å^2^. It can be seen that the simple defocused probe has almost 2 orders of magnitude worse SNR than the FZP and the random probe at the lowest spatial frequencies. At low spatial frequency the FZP achieves the best SNR, while at high spatial frequencies the random probe does slightly better. We have therefore used the random probe for the reconstructions shown in Figs 2 and 4. We give a qualitative explanation of this fact, but emphasize that a theory for optimal measurement design in ptychography and a practically feasible implementation of it is still outstanding, and may drastically improve upon the results presented here. From Fig. 5 column c) it can be seen, that the randomized probe induces the strongest intensity fluctuations (speckle) in the diffraction pattern. These very localized fluctuations vary strongly when scanning the sample, while the diffraction pattern from the defocused probe has slowly-varying features, which correlate strongly with the real space transmission function and vary only weakly when scanning the sample, which leads to a less diverse dataset and can cause problems in the reconstruction.

## Methods

### Mathematical framework of Ptychography

We define the two-dimensional grid with size ${n}_{1}\times {n}_{2}\in {\mathbb{N}}\times {\mathbb{N}}$ and length scale *r* > 0 as ${D}_{r}^{{n}_{1}\times {n}_{2}}\,:={(r\alpha ,r\beta )}_{\alpha ,\beta =0}^{{n}_{1},{n}_{2}}\subset {{\mathbb{R}}}^{2}$. The two-dimensional complex transmission function of the object is discretized as a *n*_1_ × *n*_2_ matrix and denoted as $T:{D}_{{r}_{{\rm{d}}}}^{{n}_{1}\times {n}_{2}}\to {\mathbb{C}}$, where *r*_d_ > 0 is the diffraction-limited length scale as introduced above. The object is illuminated by a small beam with known distribution, and discretized as a *m*_1_ × *m*_2_ matrix, denoted as $\psi :{D}_{{r}_{{\rm{d}}}}^{{m}_{1}\times {m}_{2}}\to {\mathbb{C}}$. For simplicity, in this paper we only consider the case *n*_1_ = *n*_2_ and *m*_1_ = *m*_2_, i.e. a uniform discretization in both axes. In the experiment, the beam is moved over the sample to positions ${\vec{r}}_{i}$, and illuminates *K* > 1 subregions to obtain *K* diffraction images. The intensity measured for position *i* is then

5 $${I}_{i}(\vec{q})={| {\mathcal F} [\psi (\vec{r}+{\vec{r}}_{i})\cdot T(\vec{r})]|}^{2},i\in \{0,\ldots ,K\},$$

where the real-space coordinates are discretized in steps of *r*_d_, and reciprocal-space coordinates in steps of (*m*_{1,2}_ *r*_d_)^−1^. Mathematically, ptychographic reconstruction can be understood as a special case of the *generalized phase retrieval problem*: given a phase-less vector of measurements ${\bf{y}}\in {{\mathbb{R}}}_{+}^{m}$ find a complex vector ${\bf{z}}\in {{\mathbb{C}}}^{n}$ such that

6 $${\bf{y}}={|{\mathscr{A}}{\bf{z}}|}^{2},$$

where ${\mathscr{A}}:{{\mathbb{C}}}^{n}\to {{\mathbb{C}}}^{m}$ is an arbitrary linear operator. We follow the notations in ref. 52 to write the ptychography problem in this form. First, we vectorize the transmission function as ${{\bf{T}}}^{V}\in {{\mathbb{C}}}^{N}$ with $N={n}_{1}\cdot {n}_{2}\in {\mathbb{N}}$. We introduce the matrix ${R}_{(i)}\in {{\mathbb{R}}}^{M\times N}$, which extracts an *m*_1_ × *m*_2_ sized area centered at position ${\vec{r}}_{i}$ from **T** ^*V*^. With these notations in place, the relation between the noise-free diffraction measurements collected in a ptychography experiment and **T** ^*V*^ can be represented compactly as

7 $${\bf{I}}={|{\bf{FQ}}{{\bf{T}}}^{V}|}^{2}={|{\bf{P}}{{\bf{T}}}^{V}|}^{2},$$

where **P** is constructed by cropping *K* regions from **T** ^*V*^ and multiplying by the incoming beam in **Q**, and applying a 2D discrete Fourier transform **F**, i.e. **P** = **FQ**:

8 $$\mathop{\overbrace{[\begin{array}{c}{I}_{1}\\ \vdots \\ {I}_{KM}\end{array}]}}\limits^{{\bf{I}}\in {{\mathbb{R}}}^{KM}}={|\mathop{\overbrace{[\begin{array}{ccc}F & \cdots  & 0\\ \vdots  & \ddots  & \vdots \\ 0 & \cdots  & F\end{array}]}}\limits^{{\bf{F}}\in {{\mathbb{C}}}^{KM\times KM}}\mathop{\overbrace{[\begin{array}{c}{\rm{d}}{\rm{i}}{\rm{a}}{\rm{g}}(\psi ){R}_{(1)}\\ \vdots \\ {\rm{d}}{\rm{i}}{\rm{a}}{\rm{g}}(\psi ){R}_{(K)}\end{array}]}}\limits^{{\bf{Q}}\in {{\mathbb{C}}}^{KM\times N}}{{\bf{T}}}^{V}|}^{2}.$$

The matrix ${\bf{P}}\in {{\mathbb{C}}}^{KM\times N}$ is sometimes called *design matrix*, because its entries determine the measurement outcome and reflect the experimental design. In the last decades many algorithms to solve this problem have been devised, only a few of which we will review with regards to low-dose reconstruction in the following section. For the subsequent analysis, we denote the *KM* row vectors of **P** as **p**_i_.

### Bayesian optimization with truncated gradients

The most prominent iterative algorithms to solve the ptychographic phase retrieval problems are the difference map (DM) algorithm^14^, and the extended ptychographic iterative engine (ePIE)^20^. The difference map belongs to the family of algorithms which use projections onto non-convex sets to reach a fix-point, i.e., the solution lying at the intersection of the two sets. It can be shown that the standard algorithm of alternating projections is equivalent to steepest-descent optimization with a Gaussian likelihood, and is not suited for low-dose reconstructions^52^, because the for this case Poisson distribution arising from discretized count events differs too strongly from a Gaussian. While this argument does not hold for the more elaborate projection algorithms like DM and relaxed averaged alternating reflections (RAAR)^58^, they also fail in practice at low doses^25, 59^, and statistical reconstruction methods have to be used. Thibault and Guizar-Sicairos^24^ have analyzed maximum likelihood methods in conjunction with a conjugate gradient update rule as a refinement step, after the DM algorithm has converged. They demonstrate improved SNR by two orders of magnitude compared to the DM algorithm alone. They note, however, that starting directly with maximum likelihood optimization often poses convergence problems.

Due to the lack of algorithms with convergence guarantees, the mathematical community has recently picked up the problem, and a host of new algorithms with provable convergence has been developed. While we do not elaborate on them here we point the interested reader to the summary articles^60, 61^ and the article^62^, which refers to the most recent developments.

Here, we focus on developments which specifically target low-dose applications. Notable in this area is the work by Katkovnik *et al*.^63^, which in addition to the maximum likelihood estimate introduces a transform-domain sparsity constraint on the object and optimizes two objective functions in an alternating fashion: one for the maximizing the likelihood, and one for obtaining a sparse representation of the transmission function. However, instead of including the Poissonian likelihood directly, an observation filtering step is performed with a Gaussian likelihood. To obtain a sparse representation of the object, the popular BM3D denoising filter is used^64^.

During the writing of this paper, Yang *et al*. suggested using the Wigner Distribution Deconvolution technique for low-dose ptychography^65^, however no statistical treatment of the measurement process is included so far.

In this work, we formulate ptychographic phase retrieval as a Bayesian inference problem, by rewriting the probability of the transmission function **T** ^*V*^ given a set of measurements ${\bf{y}}={({y}_{1},{y}_{2},\ldots ,{y}_{KM})}^{T}\in {{\mathbb{R}}}_{+}^{KM}$ according to Bayes’ rule:

9 $$P({{\bf{T}}}^{V}|{\bf{y}})=\frac{P({\bf{y}}|{{\bf{T}}}^{V})P({{\bf{T}}}^{V})}{P({\bf{y}})}.$$

Since the measurements *y*_*i*_ follow the Poissonian distribution

10 $${y}_{i}\sim {\rm{Poisson}}({I}_{i}({{\bf{T}}}^{V})),$$

the likelihood function is given by

11 $$P({\bf{y}}|{{\bf{T}}}^{V})=\prod _{i=0}^{KM}\frac{{I}_{i}{({{\bf{T}}}^{V})}^{{y}_{i}}}{{y}_{i}!}{e}^{-{I}_{i}({{\bf{T}}}^{V})}\mathrm{.}$$

The prior distribution *P*(**T** ^*V*^) is usually chosen such that it favors realistic solutions, so that noise is suppressed in the reconstructed image. Here we evaluate two different models. A simple prior, suggested in ref. 42, penalizes large gradients in the image with a Gaussian distribution on the gradient of the transmission function, which is also known as Tikhonov regularization:

12 $${P}_{{\rm{Tikhonov}}}(T)=\exp (-\,\frac{{\mu }_{0}}{\kappa }{\Vert \nabla T(\vec{r})\Vert }^{2})=\exp (-\,\frac{{\mu }_{0}}{\kappa }\sum _{i=1}^{N}{({{\rm{D}}}_{x}{{\bf{T}}}^{V})}_{i}^{2}+{({{\rm{D}}}_{y}{{\bf{T}}}^{V})}_{i}^{2})$$

with $\kappa =8\frac{{N}_{pix}^{2}}{{N}_{{\rm{m}}}{\Vert I\Vert }_{1}}$ chosen as in ref. 42. *N*_*m*_ is the total number of valid measurements, *N*_*pix*_ * *K* in the case when the detector has no hot pixels. This scales the numerical value of the prior to be close to the likelihood, such that the weight *μ*_0_ can take values between 1 × 10^−1^ and 1 × 10^−2^. D_*x*_ and D_*y*_ are the discrete forward difference operators. The second prior we evaluate is based on the work by Katkovnik *et al*.^63^ and uses sparse modeling to denoise the transmission function:

13 $${P}_{{\rm{sparse}}}({{\bf{T}}}^{V})=\exp (-\,\mu {\Vert {{\bf{T}}}^{V}-{{\bf{T}}}_{{\rm{sparse}}}^{V}\Vert }^{2})$$

Here, ${{\bf{T}}}_{{\rm{sparse}}}^{V}$ is built up by applying the BM3D collaborative filtering algorithm^64, 66^, which we briefly describe in the Supplementary Material. As input for the BM3D algorithm we transform **T** ^*V*^ into hue-saturation-value format using domain coloring. The prior *P*_sparse_(*T*) reduces the difference between the denoised version of the current transmission function and the transmission function itself. We do not take into account the marginal likelihood *P*(**y**) due to the high dimensionality of the problem. Given the likelihood function *P*(**y**|**T** ^*V*^) and the prior distribution *P*(**T** ^*V*^), we can now write the objective function for the maximum-a-posteriori (MAP) estimate:

14 $${{\bf{T}}}_{{\rm{MAP}}}^{V}\,:=\mathop{{\rm{argmin}}}\limits_{{{\bf{T}}}^{V}}{ {\mathcal L} }_{{\rm{MAP}}}({{\bf{T}}}^{V})=\mathop{{\rm{argmin}}}\limits_{{{\bf{T}}}^{V}}(-\,\mathrm{log}(\frac{P({\bf{y}}|{{\bf{T}}}^{V})P({{\bf{T}}}^{V})}{P({\bf{y}})})).$$

The log-likelihood is given as

15 $$ {\mathcal L} ({{\bf{T}}}^{V})=\sum _{i=1}^{KM}[{|{{\bf{p}}}_{i}{{\bf{T}}}^{V}|}^{2}-{y}_{i}\,\mathrm{log}({|{{\bf{p}}}_{i}{{\bf{T}}}^{V}|}^{2})],$$

and the MAP objective functions are

16 $${ {\mathcal L} }_{{\rm{Tikhonov}}-{\rm{MAP}}}({{\bf{T}}}^{V})= {\mathcal L} ({{\bf{T}}}^{V})+\frac{{\mu }_{0}}{\kappa }{\Vert \nabla T(\vec{r})\Vert }^{2}$$

and

17 $${ {\mathcal L} }_{{\rm{BM3D}}-{\rm{MAP}}}({{\bf{T}}}^{V})= {\mathcal L} ({{\bf{T}}}^{V})+{\mu }_{1}{\Vert {{\bf{T}}}^{V}-{{\bf{T}}}_{{\rm{sparse}}}^{V}\Vert }^{2},$$

for the two prior models, respectively. We calculate the gradients of both expressions:

18 $$\nabla { {\mathcal L} }_{{\rm{Tikhonov}}-{\rm{MAP}}}(T)=\sum _{i=1}^{KM}2\,{{\bf{p}}}_{i}\,{{\bf{T}}}^{V}(1-\frac{{y}_{i}}{{|{{\bf{p}}}_{i}{{\bf{T}}}^{V}|}^{2}})\,{{\bf{p}}}_{i}^{\dagger }+2\frac{{\mu }_{0}}{\kappa }\sum _{i=1}^{N}{({{\rm{D}}}_{x}{{\bf{T}}}^{V})}_{i}+{({{\rm{D}}}_{y}{{\bf{T}}}^{V})}_{i},$$

19 $$\nabla { {\mathcal L} }_{{\rm{BM3D}}-{\rm{MAP}}}(T)=\sum _{i=1}^{KM}2\,{{\bf{p}}}_{i}{{\bf{T}}}^{V}(1-\frac{{y}_{i}}{{|{{\bf{p}}}_{i}{{\bf{T}}}^{V}|}^{2}})\,{{\bf{p}}}_{i}^{\dagger }+{\mu }_{1}({{\bf{T}}}^{V}-{{\bf{T}}}_{{\rm{sparse}}}^{V})$$

Since equations (16) and (17) are non-convex functions, there is no guarantee that standard gradient descent converges to a global minimum. Recently, a non-convex algorithm for the generalized phase retrieval problem with Poisson noise was presented^67^, that provably converges to a global minimum with suitable initialization. It introduces a iteration-dependent regularization on the gradients of the likelihood to remove terms which have a negative effect on the search direction. Namely, it introduces a truncation criterion

20 $${ {\mathcal E} }^{i}({{\bf{T}}}^{V})=\{|{y}_{i}-{|{{\bf{p}}}_{i}{{\bf{T}}}^{V}|}^{2}|\le \frac{{\alpha }_{h}}{KM}{\Vert {\bf{y}}-{\bf{I}}\Vert }_{1}\frac{|{{\bf{p}}}_{i}{{\bf{T}}}^{V}|}{{\Vert {{\bf{T}}}^{V}\Vert }_{2}}\},$$

that acts on the gradient of the likelihood and suppresses the influence of measurements that are too incompatible with the reconstruction. The truncation parameter *α*_*h*_ ≥ 5 is described in ref. 67. The regularized likelihood gradient is then

21 $$\nabla { {\mathcal L} }_{{ {\mathcal E} }^{i}}({{\bf{T}}}^{V})=\sum _{i\in { {\mathcal E} }^{i}({{\bf{T}}}^{V})}^{KM}[{|{{\bf{p}}}_{i}{{\bf{T}}}^{V}|}^{2}-{y}_{i}\,\mathrm{log}({|{{\bf{p}}}_{i}{{\bf{T}}}^{V}|}^{2})]\mathrm{.}$$

We compute the next step using conjugate gradient descent^68, 69^, since this lead to much faster convergence compared to the update procedure described in ref. 67.

#### Initialization

**Figure 7:**
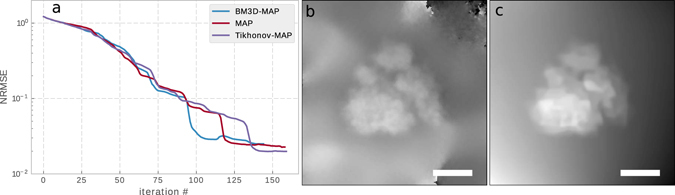
(**a**) Convergence behavior of different gradient update rules. The normalized root mean square error (NRMSE) is defined in the Supplementary material. MAP refers to a constant prior. (**b**) Example for the transmission function initialization *T* ^0^ after 70 power iterations, for an electron dose of 100 *e* ^−^/Å^2^, intensities were truncated at the 80th percentile. (**c**) ${{\bf{T}}}_{{\rm{sparse}}}^{V}$ for human ribosome after 60 iterations of BM3D-MAP. Scale bar is 10 nm.

Truncated spectral initialization for ptychography was first proposed by Marchesini *et al*.^52^, based on the notion that the highest intensities in the diffraction pattern carry the strongest phase information. They compute the phase of the largest eigenvector of the following hermitian operator:

22 $${{\bf{1}}}_{|{y}_{i}| > \varepsilon }{\bf{FQ}}{({{\bf{Q}}}^{\dagger }{\bf{Q}})}^{-1}{{\bf{Q}}}^{\dagger }{{\bf{F}}}^{\dagger }{{\bf{1}}}_{|{y}_{i}| > \varepsilon },$$

where *ε* is chosen such that the largest 20 percent of the intensities are allowed to contribute and **F** and **Q** are defined as above. The largest eigenvalue of a sparse hermitian matrix can be efficiently computed either with power iterations^70^, or with the Arnoldi method^71^. In ref. 67, truncated spectral initialization with a truncation rule with ${{\bf{1}}}_{|{y}_{i}| < {\alpha }_{0}^{2}{\lambda }_{0}^{2}}$ is used, with ${\lambda }_{0}=\sqrt{{\sum }_{i=1}^{KM}{y}_{i}}$ and *α*_0_ a free parameter. It is also important to note that the truncated spectral initialization only produces visually correct initial phase to a dose of roughly 100 *e* ^−^/Å^2^. Figure 7(b) shows an example initialization for a dose of 100 *e* ^−^/Å^2^. For doses below this value, we initialized the transmission function with unity transmission and normal-distributed phase with mean 0.1 and variance of 0.1. With this random initialization we found no problem of convergence for all algorithms tested in this paper.

#### Reconstruction parameters

All ptychography reconstructions were performed with a probe area overlap of 75% in real-space, where the probe area is defined by all pixels contributing more than 1% of the maximum intensity. This corresponds to a step size of roughly 3 nm, depending on the probe used. At a dose of 20 *e* ^−^/Å^2^, this corresponds to 8540 electrons per diffraction pattern, i.e. an average electron count of 0.52 electrons per detector pixel, and 58 electrons per image pixel for a pixel size of 1.7 Å. For the regularization parameters we performed a grid search evaluating the final normalized root mean square error and found the best values to be *μ*_0_ = 1 × 10^−2^, *μ*_1_ = 8 × 10^−2^. We choose the biorthogonal spline wavelet transform as the linear transform for BM3D as it achieves the best PSNR for high noise^72^. For the figures shown in the paper we use the BM3D denoiser. A typical intermediate denoised image for ribosome after 60 iterations can be seen in Fig. 7(c).

#### Implementation Details

The algorithms presented in this paper were implemented with the Torch scientific computing framework^73^. The gradient update routines were adapted from the optim package for Torch^69^. For efficient computing on the graphics processing unit (GPU) with complex numbers, the zcutorch library for CUDA was developed^74^. Hyperparameter optimization was done with the hypero^75^ package for Torch. For BM3D denoising we use the C++ implementation^76^. The code was run on an Intel i7-6700 processor with 32 GB RAM and a NVidia Titan X GPU with 12 GB RAM. The run time for optimization with ${ {\mathcal L} }_{{\rm{MAP}}}$ was 26 s, and for optimization with ${ {\mathcal L} }_{{\rm{BM3D}}-{\rm{MAP}}}$ 35 s. This is expected, because the BM3D algorithm used here is not yet implemented on the GPU, and the BM3D denoising is computationally more intensive.

### Data Availability

The datasets generated and analysed during the current study are available from the corresponding author on reasonable request.

## Conclusion

In this paper we have, via numerical experiments, demonstrated the feasibility of retrieving high-resolution electron transmission phase information of biological macromolecules using ptychography and Bayesian optimization. With the methods presented in this paper, it should be possible to achieve a resolution better than 1 nm for true single-particle imaging of molecular complexes with molecular weights ranging from below 100 kDa to a few MDa, and a resolution around 3 Å with simple averaging of 30 datasets. We have given a detailed explanation of the optimization and initialization procedures used, and have emphasized the importance of choosing an appropriate illumination function. We note that, while the high data redundancy in a ptychographic dataset empirically makes it experimentally very robust, there is much room for improvement in terms of measurement complexity. For the results presented here, the measurement dimension KM is larger than the problem dimension N by a factor of at least 30, while the theoretical limit for successful phase retrieval is at *KM* = 4*N* ^77^. By reducing the number of measurements, the SNR of each individual diffraction measurement could be increased, yielding an improved image SNR in the reconstruction. Therefore, the development of an optimized experimental scheme, including design of the illumination function and scanning scheme is a promising direction of research and may enable significant improvement to the results presented here.

We would like to point out two obstacles that one may have to overcome in the experimental implementation of our method. Firstly, the best results are to be expected when recording zero-loss diffraction patterns with the use of an energy filter. The energy filter may introduce phase distortions into the diffraction patterns, which may need to be accounted for in the reconstruction algorithm. Secondly, although beam-induced movements are expected to be reduced by a large amount due to spot-scanning, the remaining movement may cause problems in the reconstruction. Statistically stationary sample movements can be accounted for in the reconstruction algorithm^25, 78^, but beam-induced motions are likely to be non-stationary, and dedicated algorithms may need to be developed to account for it.

Cryogenic ptychographic imaging of biological samples is also being developed in the X-ray sciences^79^, and our results could equally be implemented there to improve the dose-effectiveness. Finally, the methods presented here may find application in electron phase imaging of radiation-sensitive samples under non-cryogenic conditions, and the incorporation of Bayesian methods into in-focus ptychographic reconstruction procedures^18, 65^, may provide similar gains in SNR as the ones discussed here, while also keeping the analytical capabilities of traditional scanning TEM imaging.

## Electronic supplementary material

Supplementary information
