# Supplementary material for: Low-dose cryo electron ptychography via non-convex Bayesian optimization
Source: Sci Rep. 2017 Aug 29;7:9883. doi: 10.1038/s41598-017-07488-y (PMC5575234; doi:10.1038/s41598-017-07488-y)
Supplement: Supplementary file 1 — Supplementary information [file 41598_2017_7488_MOESM1_ESM.pdf]

Supplementary material for: Low-dose cryo electron ptychography via non-convex Bayesian optimization

Philipp Michael Pelz[1,2,3,*], Wen Xuan Qiu[4], Robert Buecker[1,2], Guenther Kassier[1,2], and R.J. Dwayne Miller[1,2,3,4]

[1]Max Planck Institute for the Structure and Dynamics of Matter, 22761 Hamburg, Germany
[2]Center for Free Electron Laser Science, Luruper Chaussee 149, 22761 Hamburg, Germany
[3]Department of Physics, University of Hamburg, Hamburg 22761, Germany
[4]Departments of Chemistry and Physics, University of Toronto, 80 St. George Street, Toronto M5S 1H6, Canada
[*]philipp.pelz@mpsd.mpg.de

ABSTRACT

This is the supplementary material for ”Low-dose cryo electron ptychography via non-convex Bayesian optimization”

Scaling of the SNR

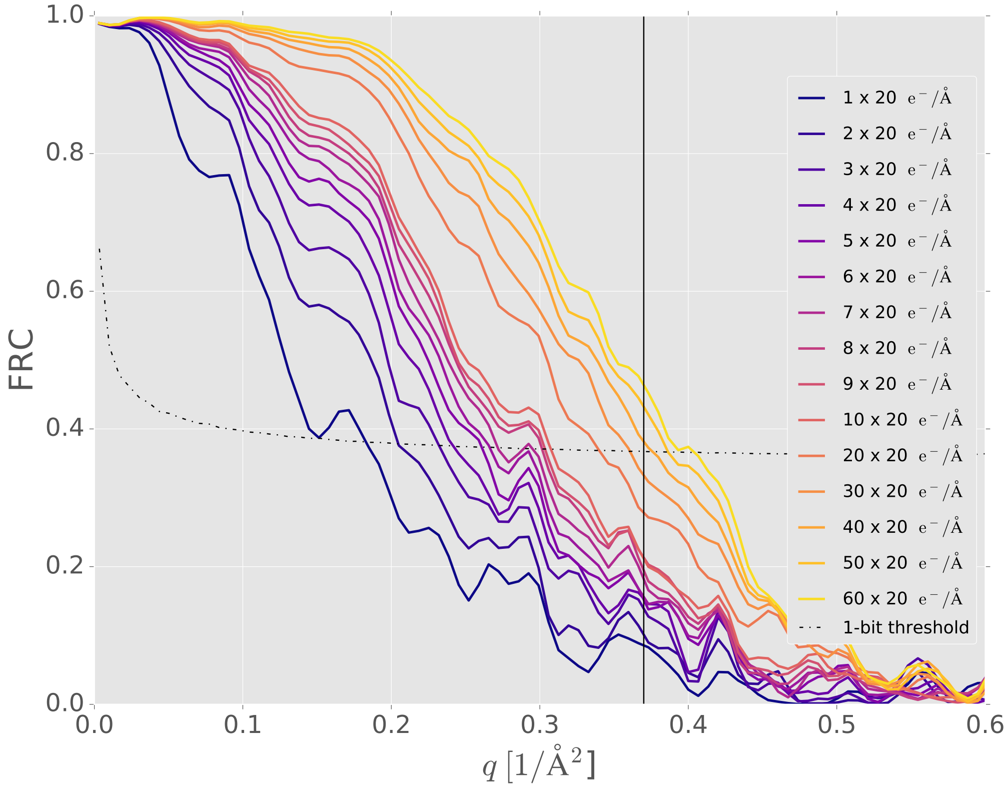

Figure 1. Scaling of the FRC between the ground truth and the averaged reconstructions with the number of averaged reconstructions for human ribosome at $20\,e^-/\text{Å}^2$. The vertical line is the resolution where the probe-forming aperture ends.

Averaged images used for the FRC calculation

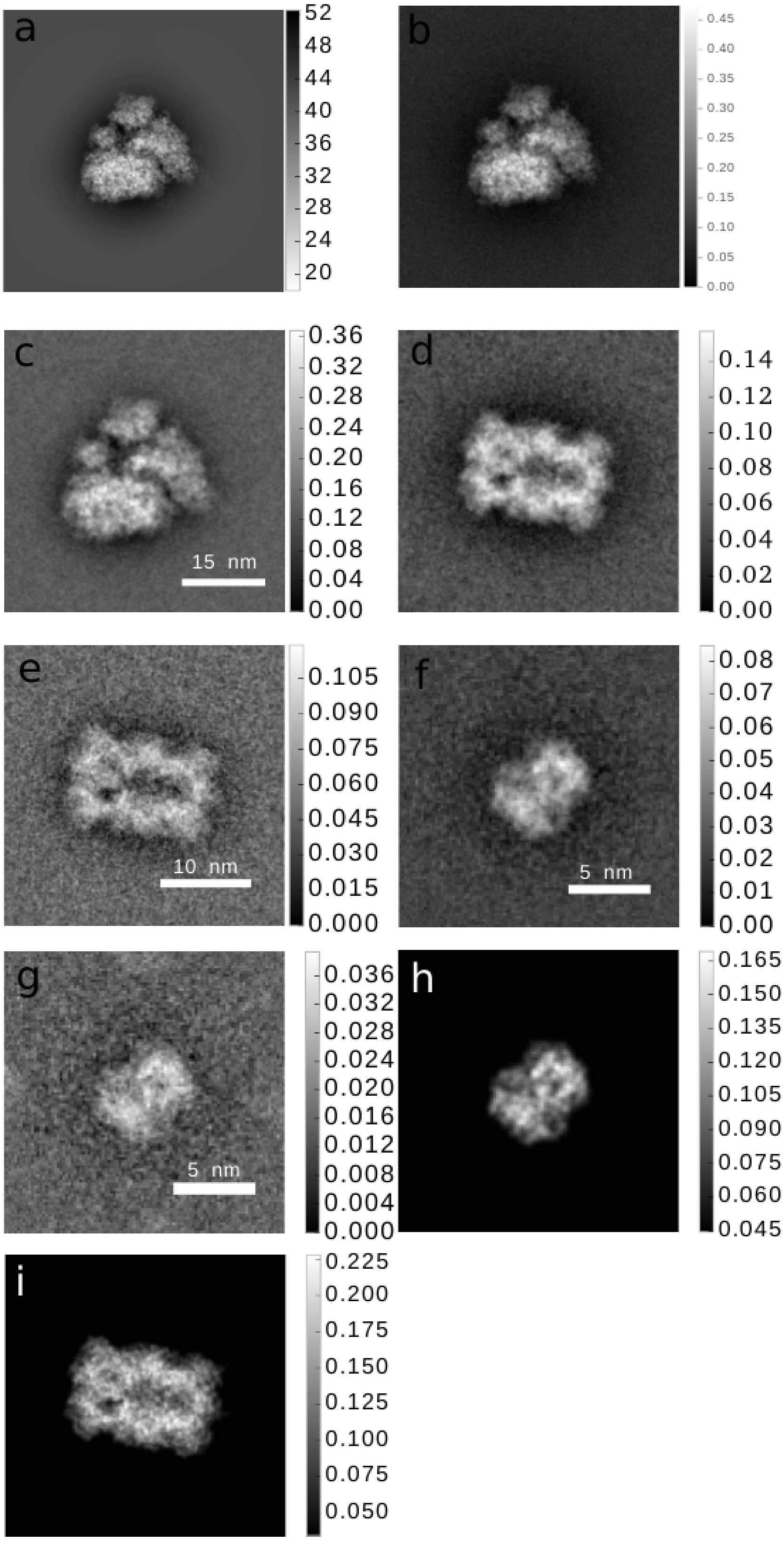

Figure 2. Images used for calculating the FRC plots in the manuscript: a) ground truth for phase-plate TEM and defocus TEM of ribosome at $20\,e^{-}/\text{Å}^2$. b) Second averaged ribosome image for average FRC calculation at $20\,e^{-}/\text{Å}^2$ c) Second averaged ribosome image for average FRC calculation at $5\,e^{-}/\text{Å}^2$ d) Second averaged proteasome image for average FRC calculation at $20\,e^{-}/\text{Å}^2$ e) Second averaged proteasome image for average FRC calculation at $5\,e^{-}/\text{Å}^2$ f) Second averaged hemoglobin image for average FRC calculation at $20\,e^{-}/\text{Å}^2$ g) Second averaged hemoglobin image for average FRC calculation at $5\,e^{-}/\text{Å}^2$ h) ground truth for phase-plate TEM and defocus TEM at $20\,e^{-}/\text{Å}^2$

Normalized root mean square error

The normalized root mean square error is defined as

$$NMSR = \min_{\xi \in \{\xi \in \mathbb{C}:|\xi|=1\}} \frac{||\xi \mathbf{T} - \mathbf{T}_{model}||_2}{||\mathbf{T}||_2} \quad (1)$$

Brief explanation of BM3D filtering

The BM3D algorithm computes the transform-domain sparse representation in four steps: 1) it finds the image patches similar to a given image patch and groups them in a 3D block 2) 3D linear transform of the 3D block; 3) shrinking of the transform spectrum coefficients; 4) inverse 3D transformation

Discussion of phase retrieval algorithms

The PIE algorithm can be formulated as maximum-a-posteriori (MAP) optimization with a Gaussian likelihood function, and an independently weighted Gaussian prior of the object change [1, 2], combined with a stochastic gradient-like update rule. The noise performance of the PIE algorithm has been investigated in [1], and found to be worse than the Poissonian likelihood model at low counting statistics. While practically very robust, both algorithms can get stuck in local minima and until recently [3], no proof of convergence to a global minimum existed. In [1] it is suggested to use a global gradient update at the start to avoid stagnation, and [4] use a restarted version with the stochastic gradient update rule, after removal of phase vortex artifacts. Another recent, similar approach uses dictionary learning to obtain a sparse representation of the transmission function [5], however, only real-valued signals are treated.

References

1. Godard, P., Allain, M., Chamard, V. & Rodenburg, J. Noise models for low counting rate coherent diffraction imaging. *Opt. Express* **20**, 25914–34 (2012). URL http://www.ncbi.nlm.nih.gov/pubmed/23187408. 00024.

2. Thibault, P. & Menzel, A. Reconstructing state mixtures from diffraction measurements. *Nat.* **494**, 68–71 (2013). DOI 10.1038/nature11806. 00135.

3. Marchesin, S., Tu, Y. & Wu, H.-t. Alternating Projection, Ptychographic Imaging and Phase Synchronization. *arXiv Prepr. arXiv1402.0550* 1–29 (2014). URL http://arxiv.org/abs/1402.0550. 00000, arXiv:1402.0550v1.

4. Maiden, A. M., Sarahan, M. C., Stagg, M. D., Schramm, S. M. & Humphry, M. J. Quantitative electron phase imaging with high sensitivity and an unlimited field of view. *Sci. Reports* **5**, 14690 (2015). DOI 10.1038/srep14690. 00005.

5. Tillmann, A. M., Eldar, Y. C. & Mairal, J. DOLPHIn #x2014;Dictionary Learning for Phase Retrieval. *IEEE Transactions on Signal Process.* **64**, 6485–6500 (2016). DOI 10.1109/TSP.2016.2607180. 00000.
